# Roles of the Hedgehog Signaling Pathway in Epidermal and Hair Follicle Development, Homeostasis, and Cancer

**DOI:** 10.3390/jdb5040012

**Published:** 2017-11-20

**Authors:** Yoshinori Abe, Nobuyuki Tanaka

**Affiliations:** Department of Molecular Oncology, Institute for Advanced Medical Sciences, Nippon Medical School, 1-396 Kosugi-machi, Nakahara-ku, Kawasaki 211-8533, Japan; yoshiabe@nms.ac.jp

**Keywords:** Hedgehog signaling, epidermis, hair follicle, development, homeostasis, basal cell carcinoma, signaling crosstalk

## Abstract

The epidermis is the outermost layer of the skin and provides a protective barrier against environmental insults. It is a rapidly-renewing tissue undergoing constant regeneration, maintained by several types of stem cells. The Hedgehog (HH) signaling pathway is one of the fundamental signaling pathways that contributes to epidermal development, homeostasis, and repair, as well as to hair follicle development and follicle bulge stem cell maintenance. The HH pathway interacts with other signal transduction pathways, including those activated by Wnt, bone morphogenetic protein, platelet-derived growth factor, Notch, and ectodysplasin. Furthermore, aberrant activation of HH signaling is associated with various tumors, including basal cell carcinoma. Therefore, an understanding of the regulatory mechanisms of the HH signaling pathway is important for elucidating fundamental mechanisms underlying both organogenesis and carcinogenesis. In this review, we discuss the role of the HH signaling pathway in the development and homeostasis epidermis and hair follicles, and in basal cell carcinoma formation, providing an update of current knowledge in this field.

## 1. Introduction

Although the thickness of the skin is as little as 2 mm on average, it is a complex stratified organ consisting of an outer epidermis and appendages (for example, hair follicles, sebaceous glands, and sweat glands) separated from the underlying dermis by a basement membrane. It is the largest organ in the human body, by area and weight. Skin is the primary barrier that protects the body from major environmental stresses, such as dehydration, scratches, wounds, and microbial infection [1]. To repair damaged tissue and replace old cells, different stem cell pools resident in the skin contribute to the maintenance and repair of the various epidermal tissues, including interfollicular epidermis, hair follicles, sebaceous glands, and sensory touch domes [2,3]. The stem cells residing in the epidermis are maintained by various signaling pathways such as those activated by Wnt, transforming growth factor β (TGF-β), Notch, and Hedgehog (HH) [3]. These signaling pathways also have important roles in embryonic skin development [4]. However, the dysregulation of signaling pathways related to skin development and homeostasis induces epigenetic reprograming of skin cells, resulting in cancer development. In particular, aberrant activation of HH signaling is the major cause of basal cell carcinoma (BCC) [5]. Studies of tumorigenesis mediated by HH signaling activation have recently culminated in the USA Food and Drug Administration’s (FDA) approval of GDC-0449 (vismodegib) [6], an oral inhibitor of the HH signaling mediator, Smoothened (SMO), as a therapeutic for treating advanced or metastatic BCC. In this review, we provide a current understanding of the role of the HH signaling pathway in embryonic skin development, skin homeostasis, and skin cancer.

## 2. The HH Signaling Pathway

**Figure 1:**
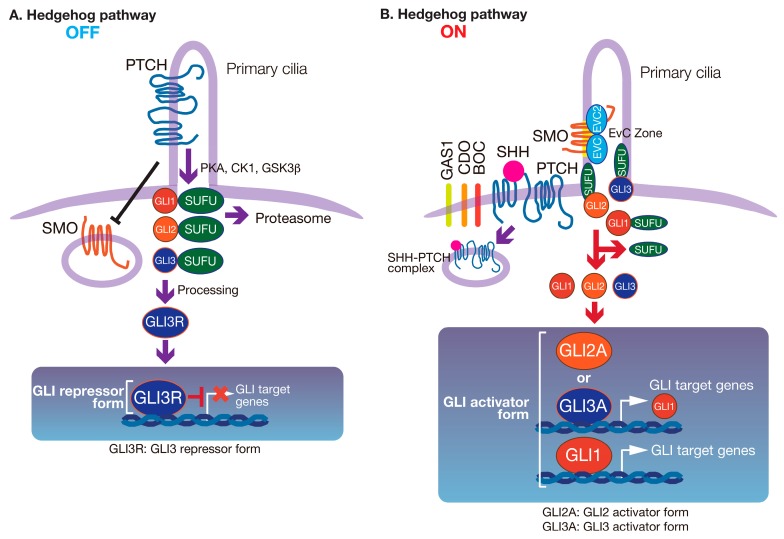
The Hedgehog signaling pathway. (**A**) In the absence of Hedgehog (HH) ligand, Patched (PTCH) blocks the ciliary localization of Smoothened (SMO), and a repressor form of GLI (mainly GLI3R) suppresses the induction of GLI target genes. (**B**) Signaling is activated in the presence of HH ligand. The binding of HH ligand to PTCH prevents PTCH inhibition of SMO, and SMO then interacts with the Ellis van Creveld Syndrome (EVC)–EVC2 complex and translocates into primary cilia to be fully activated. SMO then activates GLI family transcription factors, mainly GLI2. GLI2 up-regulates the expression of GLI1, as well as GLI target genes. GLI1 is also activated downstream of SMO. Activated GLI2 (GLI2A) and GLI1 further upregulate the expression of various GLI target genes. This figure has been adapted from our previous work [20].

The HH pathway is one of the most important signal transduction pathways in tissue development, homeostasis, and repair. It regulates the morphogenesis of various organs during embryogenesis [7]. We show a schematic diagram of the HH signaling pathway in Figure 1. However, HH signaling is complex, and several comprehensive reviews have been published describing the molecular mechanisms in detail [8,9,10,11]. In the canonical pathway, HH signaling is initiated by one of three spatiotemporally-onfined ligands: Sonic Hedgehog (SHH), Indian Hedgehog (IHH), and Desert Hedgehog (DHH). HH ligands control numerous developmental outcomes in a concentration- and duration-dependent manner. Each HH ligand has distinct spatial and temporal expression patterns, but all activate HH signaling by binding to Patched (PTCH) 1 or 2, which are 12-pass transmembrane-spanning receptors. PTCH1 is considered to be the primary receptor for HH ligands. In the absence of HH, PTCH1 is localized in primary cilia and constitutively suppresses the activity of SMO, a seven-pass transmembrane-spanning protein that is a member of the G-protein-coupled receptor superfamily [12]. Suppressor of fused (SUFU) is a key negative regulator of the HH signaling pathway [13]. In the absence of HH ligands, SUFU inhibits signaling by sequestration of GLI proteins in the cytoplasm (Figure 1A). In addition to PTCH, other HH-binding cell surface proteins have been identified, such as CAM-related/down-regulated by oncogenes (CDO), brother of Cdo (BOC) and growth-arrest-specific 1 (GAS1) [14,15]. These molecules function as HH co-receptors to facilitate HH signal reception. Following the binding of a HH ligand to PTCH, SMO accumulates at a specialized compartment in the primary cilium, called the EvC zone, mediated by the Ellis van Creveld Syndrome (EVC)–EVC2 complex [16,17]. From here, SMO transmits signals that activate GLI (Figure 1B) [18,19].

Graded levels of HH signaling trigger the expression of different sets of response genes, depending on the ratio of GLI activator (GLI-A) to repressor (GLI-R) forms. In vertebrates, the GLI family consists of three proteins, GLI1, GLI2, and GLI3 [10]. All GLI proteins contain an activator domain at their C-terminus; GLI2 and GLI3 also have an N-terminal repressor domain [21]. Studies in mutant mice suggest that GLI2 is the major activator of HH signaling [22], whereas GLI3 is the major repressor [23,24]. GLI1 most likely serves as a signal amplifier downstream of GLI2 [22,25]. When HH signaling is activated, SUFU–GLI2 complexes dissociate and GLI2 is activated [26]. *Gli2* knockout mice die at birth, whereas *Gli1* knockout mice show normal development, unless one copy of *Gli2* is also defective [27]. Interestingly, experiments in mutant mice further suggest that Gli2 can rescue Gli1 protein function, whereas *Gli1* knock-in into the *Gli2* allele can rescue the *Gli2*-null phenotype [28].

Constitutive activation of HH signaling has been observed in many cancers [29] and promotes cancer cell proliferation, metastasis, and cancer stem cell maintenance. For example, somatic mutations of *PTCH1* and *SMO* have been identified in patients with BCC and medulloblastoma [30,31,32,33]. Other mutations in genes encoding HH pathway components have been reported, including *SUFU* mutations in BCC and medulloblastoma [34]. Furthermore, multiple mechanisms of HH pathway activation in cancer have been proposed, and reviewed in detail elsewhere [10,11,35].

HH signaling exhibits crosstalk with other signaling pathways, including Wnt [36,37,38,39], TGF-β [40], epidermal growth factor receptor (EGFR) [41], rat sarcoma viral oncogene homolog GTPase (RAS)–extracellular signal-regulated kinase (ERK) [42,43], phosphatidylinositol 3-kinase (PI3K)–Akt–mechanistic target of rapamycin (mTOR) [44], and Notch [45]. Interestingly, the crosstalk between HH signaling and other pathways seem to be different between developmental, homeostatic, and carcinogenic processes.

## 3. Overview of Epidermal and Hair Follicle Development

### 3.1. Development of the Epidermis

During embryonic development, the epidermis is derived from the ectoderm. In mice, after gastrulation, the embryo surface consists of a single layer of neuroectoderm, which will form the nervous system and skin epithelium from embryonic day 9.5 (E9.5) to E12.5. Mesenchymal cells from the underlying layer transmit signals that instruct the stratification of the epidermis and dictate the positioning of downgrowths that mark the initiation of hair follicle morphogenesis [1]. In alliance with the mesenchyme, the inner-most, or basal layer of the stratifying epidermis produces and organizes an underlying basement membrane that is rich in extracellular matrix proteins and growth factors. The epidermis adheres to this basement membrane, which serves not only as a growth-promoting platform but also as a physical boundary between the epithelium and the dermis [46]. During the initial steps of stratification (E12.5–E15.5), cell proliferation is almost completely confined to the basal layer, and these cells differentiate immediately. In this period, the single layer of epidermal cells is covered by a transient protective layer called the periderm. Although there is regional variation, the stratification program is largely complete around E17.5 [47].

### 3.2. Hair Follicle Development

**Figure 2:**
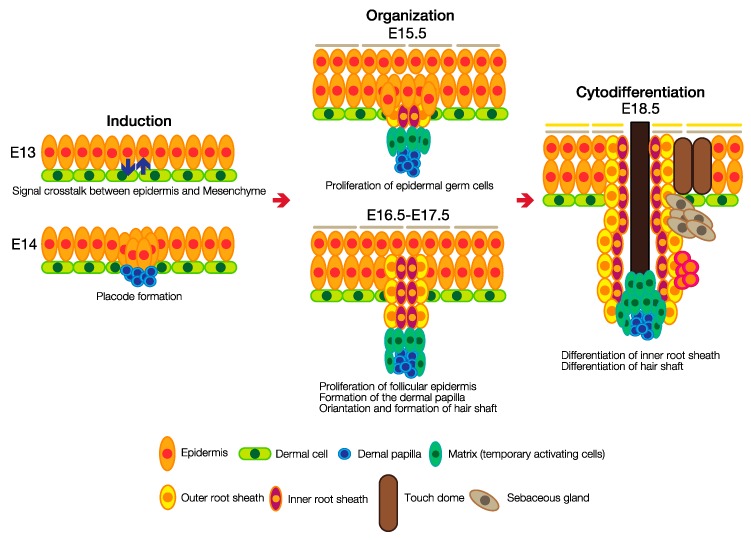
At the beginning of hair follicle development, gradients of activators and inhibitors generate a well-circumscribed inductive field (E13). These gradients induce epithelial and dermal cell proliferation to develop a placode and a dermal papilla (E14). The dermal papilla sends growth signals back to the epithelial cells to form the hair follicle (E15.5–E17.5). In the cytodifferentiation phase, epithelial cells differentiate into inner and outer root sheath, which generates the hair shaft. In addition, melanocytes and hematopoietic cells migrate into the hair follicle, and touch domes and sebaceous glands are formed.

The development of hair follicles is intrinsically related to the stratification of the embryonic epidermis. Hair follicle development is the result of epithelial–mesenchymal interactions. Although hair follicle development occurs in eight morphologically distinct stages, it can more simply be divided into three phases: induction, organogenesis and cytodifferentiation [48]. In the induction phase, which is the initiation of hair follicle development (E13), signaling crosstalk between the epidermis and dermis is induced, and epidermal keratinocytes form clusters that become enlarged and elongated to form hair placodes (E14). Hair follicle development then enters the organogenesis phase. A cluster of specialized fibroblasts, the precursors of the dermal papilla, is formed just under the placode, and the signaling crosstalk between these specialized fibroblasts and the epithelial placode leads to cell proliferation in both structures. The enhanced cell proliferation leads to a downward growth of the epidermal component, shaping the dermal papilla. The resulting structure, called the hair germ, is typically observed at E15.5. Keratinocyte downgrowth continues, whereby the most proximally located keratinocytes begin to wrap around the dermal papilla (E16.5–E17.5). As follicles enter the cytodifferentiation phase, the inner root sheath is formed, triggering the terminal differentiation of keratinocytes above the dermal papilla to generate the hair shaft. Simultaneously, the outer root sheath starts to form a cylinder around the inner root sheath to create a bulbous peg structure (E18.5). Additional appendages such as sebaceous glands and touch domes also develop during this phase. These timelines are summarized in Figure 2. For a more detailed discussion of hair follicle development, please refer to several published reviews [2,4,49].

## 4. The Roles of HH Signaling in Hair Follicle and Touch Dome Development

In mouse skin at E15.5, at the beginning of the organogenesis phase of hair follicle development, *Ptch1*, *Smo*, *Gli1* and *Gli2* are expressed in both the epithelial and dermal components of the early hair follicle, although higher levels of *Ptch1* and *Gli1* are observed in the follicular dermal mesenchyme. In contrast, Ptch2 expression is restricted to epithelial cells of the hair bud where it is co-expressed with *Shh* [47,50]. Therefore, the HH signaling pathway functions in both the epithelium and the mesenchyme. In mice lacking *Shh*, hair follicle formation is initiated and specialized fibroblasts are condensed, indicating that the initial events of hair follicle development are independent of HH signaling. However, in the hair follicle maturation phase, the loss of *Shh* selectively reduces keratinocyte proliferation in the developing follicle epithelium, so that the downgrowth of epithelial cells fails. Therefore, the number of hair follicles is reduced in *Shh*^−/−^ mice. Thus, HH signaling is necessary for the transition from the induction to the organogenesis phase, and for the downgrowth of epithelial cells after the induction phase. Furthermore, the maturation of the dermal papilla also depends on HH signaling, since *Shh*^−/−^ mice have smaller dermal papillae [50,51]. It is believed that GLI2 is the primary transducer of HH signaling, whereas GLI1, the expression of which is transcriptionally regulated by GLI2, plays a secondary role in potentiating the HH response [10]. Mill et al. demonstrated that hair follicle maturation fails in *Gli2*^−/−^ skin because of suppressed epithelial cell proliferation, similar to *Shh*^−/−^ skin [52]. Development of the dermal papilla is also severely affected by the failure to respond to SHH in *Gli2*^−/−^ skin. In addition, *Shh*^−/−^, as well as *Gli2*^−/−^, skin fails to induce GLI1. Together, these results show that the SHH–GLI2 axis is required for the proliferation and subsequent downgrowth of the follicular epithelium, and for the maturation of the dermal papilla.

As described above, the expression of *Ptch1*, *Smo*, *Gli1*, and *Gli2* is seen in the dermal papilla [50], indicating that HH signaling also functions in dermal cells. RNAi-mediated SMO knockdown in dermal cells results in the loss of the dermal papilla precursor, the dermal cell condensate, and in hair follicle arrest at the organogenesis phase, similar to *Shh*^−/−^ skin. However, HH signaling in dermal cells does not affect cell survival or epithelial proliferation. Rather, dermal HH signaling regulates the expression of a subset of dermal papilla-specific signature genes. These results suggest that dermal HH signaling regulates genes required to maintain the maturation of the papilla [53]. Sex-determining region Y-box 2 (SOX2) is expressed only in the dermal papilla and determines hair follicle type [54,55]. Recently, Driskel et al. demonstrated that dermal papilla cells are heterogenous and SOX2^+^/CD133^+^ dermal papilla cells are associated with guard, awl, and auchene hair follicle formation whereas SOX2^−^/CD133^+^ dermal papilla cells are involved in the formation of zigzag hair follicles. Moreover, they demonstrated the HH signaling pathway is activated in SOX2^−^/CD133^+^ dermal papilla cells, suggesting that HH signaling regulates hair type fate determination through the maintenance of dermal papilla cell heterogeneity [55].

**Figure 3:**
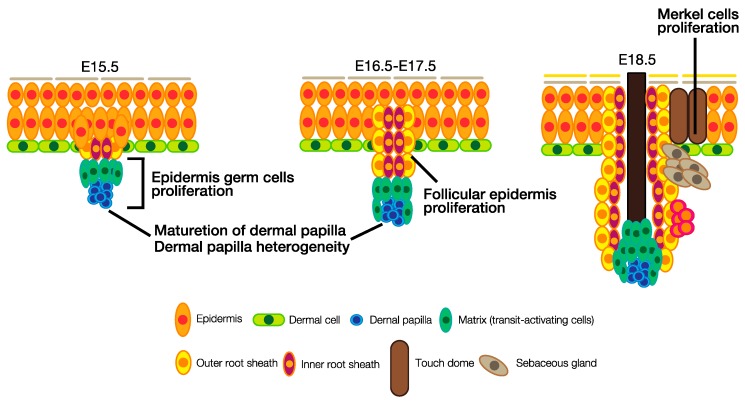
The roles of the HH signaling pathway in embryonic hair follicle development. In epidermal cells, HH signaling is involved in germ cell and follicular cell proliferation. HH signaling is also involved in the maturation of the dermal papilla, as well as dermal papilla heterogeneity. Furthermore, HH signaling is essential for Merkel cell proliferation and touch dome development.

Touch domes are unique epidermal sensory structures that form exclusively around primary hair follicles [56,57]. Merkel cells are the mechanoreceptors found in touch domes. Merkel cells in the epidermis can be identified based on their expression of atonal bHLH transcription factor 1 (ATOH1), SOX2, and Keratin 8 [58]. Recently, it has been revealed that the hair follicle functions as a niche required for Merkel cells formation. In *Shh*-null skin, Merkel cells are absent, and SHH overexpression results in increased proliferation of Merkel cells. HH signaling pathway activation, which is required for Merkel cell formation, is initiated by the production of SHH in developing hair follicles. Therefore, HH signaling pathway is required for Merkel cell formation, resulting in touch dome development [59,60]. The roles of the HH signaling pathway in hair follicle and touch dome development are summarized in Figure 3.

On the other hand, negative regulators of HH signaling are also important for hair follicle development. PTCH1 and 2 are known to be negative regulators of HH signaling, and Ptch1 is a GLI target gene [47,61]. Basal cell-specific *Ptch1*-null skin exhibits hyperplasia and skin neoplasia formation, although *Ptch2*-null skin is undistinguishable from wild-type skin with respect to embryonic hair follicle development [62,63]. However, the skin of both basal cell-specific *Ptch1*-null and *Ptch2*-null mice exhibits excessive proliferation of epithelial cells, suggesting that epidermal stem and progenitor cells enter a hyperproliferative state. In addition, progenitor cells fail to differentiate, resulting in the loss of epidermal lineage specification. Therefore, PTCH1- and 2-mediated HH signaling suppression is important for regulating epidermal differentiation [64]. Another study reported that increased numbers of immature hair follicles in the induction phase are seen in *Shh*^−/−^/*Gli3*^−/−^ skin, in which the repressor form of GLI3 (GLI3R), another negative regulator of HH signaling, is lost. Furthermore, keratinocyte differentiation fails in *Shh*^−/−^/*Gli3*^−/−^ skin, resulting in the failure of hair follicle maturation [65]. Recently, Adolphe et al. discovered that there is a HH signaling gradient along the proximodistal axis of developing hair follicles [66]. At E17.5, the peak of HH signaling activity is in follicular cells, which are a source of SHH, and HH signaling is endogenously low in basal cells. This HH signaling gradient is established by PTCH1 and PTCH2. These results suggest that HH signaling has the capacity for progenitor cell maintenance, and the intensity of HH signaling is important for both epithelial and mesenchymal cell fate determination during hair follicle development.

## 5. Signaling Crosstalk between HH and Other Pathways in Hair Follicle Development

The hair follicle develops as a result of complex reciprocal signaling between epithelial and mesenchymal cells. Various signaling pathways, such as those activated by Wnt, bone morphogenetic protein (BMP), platelet-derived growth factor (PDGF), Notch and ectodysplasin, are involved in hair follicle development, and exhibit signaling crosstalk with the HH pathway. In this section, we discuss the crosstalk between HH and these other signaling pathways in hair follicle development.

### 5.1. Wnt Signaling

The Wnt signaling pathway has been implicated in many developmental processes [67]. Canonical Wnt signaling is mediated by a transcriptionally active complex between stabilized β-catenin and members of the T-cell factor (TCF)/Lymphoid enhancer-binding factor 1 (LEF-1) family of DNA-binding proteins [68]. One of the first, essential events required to form a follicle placode is the activation of Wnt signaling in the epidermis. Wnt signaling also transmits subsequent signals for hair follicle development and patterning. Wnt ligands are expressed in interfollicular epidermis as well as the hair follicle during all stages of follicle development, suggesting that Wnt signaling is a master regulator of follicle development [36,69,70].

The Wnt and HH signaling pathways are often associated. During hair follicle development in mouse embryos, the canonical Wnt signaling pathway is upstream of HH signaling, since β-catenin activation induces Shh expression in epidermis during skin development [36,37]. On the other hand, Reddy et al. showed that dermal Wnt5a disappears in *Shh*^−/−^ skin, indicating that Wnt5a is downstream, or even a direct target, of HH signaling [71]. Therefore, SHH-induced dermal Wnt5a, as well as GLI1 expression, is important for dermal papilla maturation. Wnt5a suppresses expression of LEF-1, a well-recognized Wnt/β-catenin signaling target in human dermal papilla cells, indicating suppression of canonical Wnt signaling [72]. Therefore, the HH–Wnt5a axis terminates cell proliferation and induces differentiation to promote mature hair follicle formation. Since PTCH1 and 2, repressors of HH signaling, are also involved in the induction of cell differentiation, cooperation between Wnt5a and negative regulators of HH signaling may be important for cell differentiation during hair follicle development.

### 5.2. BMP Signaling

BMPs are members of TGF-β family. BMP signaling has been suggested to regulate hair follicle induction and the patterning of follicles within the skin by repressing the placode fate [73,74]. Suzuki et al. demonstrated crosstalk among the Wnt, HH, and BMP signaling pathways during hair follicle development [37]. Expression of constitutively active β-catenin induces aberrant keratinocyte proliferation and differentiation in mouse skin during hair follicle development; the aberrant cell differentiation is suppressed by BMP signaling inhibition, and the aberrant cell proliferation is suppressed by HH signaling inhibition. These results demonstrate that Wnt/β-catenin signaling relayed through HH and BMP signals is the principal regulatory mechanism underlying changes in hair follicle cell fate. Furthermore, activation of the HH signaling pathway induces expression of BMP2 in the organogenesis phase of hair follicle development. A possible mechanism for the regulation of hair follicle development involves cell fate being controlled by the difference in the diffusion area between promoters, such as SHH, and suppressors, such as BMP [75,76]. Therefore, the presence of signaling crosstalk involving the Wnt, HH, and BMP pathways would regulate the diffusion areas of HH and BMP ligands to determine hair follicle cell fate.

The HH signaling pathway also affects dermal cells during hair follicle development. Noggin is known as an antagonist of BMPs, and Noggin signaling is a dermal papilla-specific pathway. In *noggin*^−/−^ mice, hair follicles arrest at the placode formation stage of embryonic development [73,77]. Oro et al. showed that interaction between HH signaling and Noggin regulates dermal papilla maturation during hair follicle development [53]. Dermal cell-specific RNAi-mediated SMO knockdown does not affect dermal cell proliferation in mice. However, dermal cell condensation following placode formation fails in these mice. These authors found that dermal HH signaling induces dermal papilla signature genes such as *Sox2* [54,55], *Sox18* [78,79], and *noggin*. Among these genes, noggin partially rescues dermal SMO knockdown-associated hair regeneration defects by increasing epithelial *Shh* expression. These results suggest that dermal HH signaling regulates specific dermal papilla gene expression signatures to maintain dermal papilla maturation while maintaining a reciprocal HH–Noggin signaling loop to drive embryonic hair follicle development.

### 5.3. PDGF Signaling

A role for PDGF-A in hair follicle development has been suggested by the injection of PDGF-receptor α (PDGFRα) antibodies into newborn mice, which perturbed hair formation [80]. Karlsson et al. demonstrated the roles of crosstalk between PDGF and HH signaling in hair follicle development [81]. *pdgf-a*-null skin exhibits thinner dermis, smaller dermal papillae and abnormal hair follicle shape. *Shh* and *pdgf-a* expression overlap during hair follicle development. Moreover, PDGFRα is expressed in dermal cells adjacent to sites of SHH expression (unpublished data referred to in [81]). In *Shh*-null skin, the aggregation of PDGFRα-expressing dermal cells beneath the epithelial cells fails, implying the failure of dermal papilla formation [81]. As described in Section 5.2, HH signaling upregulates noggin expression in dermal cells to maintain dermal papilla maturation. Gao et al. reported that HH signaling together with PDGF signaling promotes the expression and secretion of Noggin in mesenchymal cells [82]. These results suggest that the cooperation of HH and PDGF signaling is necessary for dermal papilla formation during hair follicle development.

### 5.4. Notch Signaling

Notch signaling plays a crucial role in cell fate determination and developmental border formation [83]. During epidermal development, Notch signaling regulates the process of commitment to either the hair follicle or the interfollicular epidermis lineage [84]. Furthermore, Notch signaling terminates the differentiation program that culminates in skin barrier formation [85]. As described above in Section 4, HH signaling is involved in the maintenance of SOX2^−^/CD133^+^ dermal papilla cells [55]. The Notch signaling pathway is also activated in these cells [55]. Previous studies demonstrated that Notch signaling induces SHH [45] and GLI2 [86,87] expression in stem cells. Therefore, Notch signaling may regulate HH signaling for the maintenance of dermal papilla cell heterogeneity.

### 5.5. The EdaA1–EdaR–NF-κB Axis

Ectodysplasin A1 (EdaA1) and ectodysplasin receptor (EdaR) respectively belong to the tumor necrosis factor (TNF) multigene family of ligands and receptors, and are able to activate the nuclear factor-κB (NF-κB) and Jun amino-terminal kinase (JNK)/activator protein 1 (AP1) pathway in vitro [88,89,90,91]. The EdaA1–EdaR pathway is activated at placode formation only in primary guard hair follicles. It has been reported that the EdaA1–EdaR–NF-κB axis is involved in placode formation, since NF-κB is directly activated by EdaA1–EdaR signaling via the IκB kinase (IKK) complex. Schmidt-Ullrich et al. demonstrated an interaction between the EdaA1–EdaR–NF-κB axis and the HH signaling pathway [92]. They found that EdaA1–EdaR signaling-mediated NF-κB activation is essential for the induction of SHH expression and subsequent placode downgrowth. However, NF-κB induces cyclin D1 indirectly, suggesting direct induction by HH (or Wnt) signaling. These results, taken together, suggest that, during hair follicle development, NF-κB transmits EdaA1–EdaR signaling to activate the HH signaling pathway via the direct NF-κB-mediated induction of SHH.

## 6. Epidermal Homeostasis

The adult epidermis consists of several layers, each with its own characteristics. Cells in the basal layer proliferate to provide new cells that replace those shed from the cornified exterior of the tissue [93]. This renewal process is called homeostasis. Epidermal homeostasis requires a balance between keratinocyte proliferation and differentiation [94,95]. In the skin, multiple populations of epidermal stem cells have a crucial role in maintaining tissue homeostasis, providing new cells to replace those that are constantly lost during tissue turnover or following injury [96]. The epidermis is composed of the interfollicular epidermis, hair follicles, sebaceous glands and sweat glands, and each of these tissues has its resident stem cells. The permanent, lower part of the hair follicle, known as the bulge, is one reservoir of stem cells. Bulge stem cells were originally identified as slow cycling cells [97,98]. A large number of markers have been described for these cells. CD34 cell surface glycoprotein [99,100] and Leu-rich repeat-containing G protein-coupled receptor 5 (LGR5) are considered to be markers of bulge stem cells [101,102]. Recent studies have reported that HH signaling is involved in the maintenance of one type of bulge stem cells. We discuss the role of the HH signaling pathway in bulge stem cell maintenance below.

## 7. Roles of the HH Signaling Pathway in Epidermal Homeostasis

Treatment of adult mice with an anti-SHH antibody blocks the active phase of the hair growth cycle (anagen) and blocks hair regrowth [103], indicating that SHH is required for the regenerative function of adult bulge stem cells. Conversely, exogenously-administered SHH triggers anagen onset in follicles in the resting phase of the hair cycle (telogen), and stimulates hair growth [104]. These reports suggest that HH signaling is involved in hair follicle maintenance.

In adult skin, stem cells in the hair follicle bulge cyclically regenerate the follicle, whereas a distinct stem cell population maintains the epidermis. In telogen hair follicles, GLI2 and GLI3 continue to be broadly expressed in both the follicle and the surrounding dermis. Throughout the hair cycle, GLI1 expression is restricted to two distinct epidermal stem cell domains [105]. One population of GLI1-expressing (GLI1^+^) stem cells co-expresses LGR5 [101,102]. Stem cells expressing both GLI1 and LGR5 (GLI1^+^/LGR5^+^) exhibit greater proliferative properties and are localized to the lower portion of the bulge, close to the telogen hair germ. In this phase of the hair cycle, GLI1^+^/LGR5^+^ stem cells only partially overlap with other bulge stem cell markers, such as CD34 and keratin 15. However, in anagen hair follicles, GLI1^+^/LGR5^+^ stem cells are localized to the lower part of the outer root sheath of the reforming hair follicle, where they lose expression of the bulge stem cell markers [106]. This localization change during the hair cycle suggests that GLI1^+^/LGR5^+^ stem cells may also be necessary for hair follicle growth in anagen. Another population of GLI1^+^ stem cells in the dermal papilla is involved in dermal papilla maintenance. These results show that, during anagen, the induction of HH target genes, including *Gli1*, occurs in both epidermal and dermal papilla cells adjacent to SHH-expressing cells, such as those in the follicle matrix.

**Figure 4:**
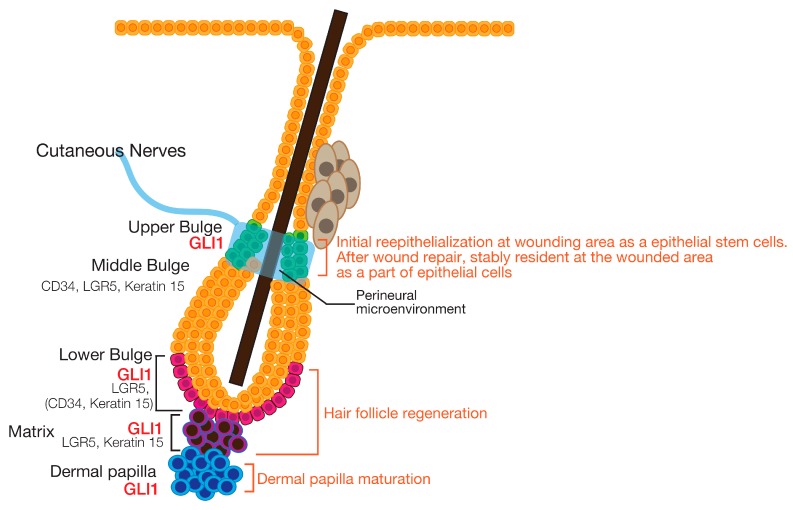
GLI1-expressing hair follicle stem cells and their roles. The expression of GLI1 and other markers define molecularly distinct zones in the telogen hair follicle, including regionalization of the bulge into the upper, middle, and lower bulge. GLI1-expressing cells in the upper bulge receive a HH signal from hair follicle-associated nerve cells, and are functionally distinct from cells in the middle bulge, lower bulge, and matrix in their ability to become epidermal stem cells during wound repair. The roles of each GLI1^+^ bulge stem cell population are described in orange text on the right.

Another population of GLI1^+^ stem cells that does not express LGR5, CD34, or keratin 15 exists in the upper fringe of the bulge. GLI1^+^ upper bulge stem cells are induced by bulge­associated nerve cells releasing SHH. GLI1^+^ stem cells from the upper bulge stably remain in a wounded area of the skin long after it has been repaired [105]. Therefore, upper bulge cells might constitute a long-term reservoir of stem cells poised to contribute to the repair of the interfollicular epidermis in a permanent manner. Interestingly, the HH signaling pathway is essential for the contribution of upper bulge stem cells to wound repair, but not for hair follicle cycling. This emphasizes the relevance of the niche for the function of different populations of hair follicle stem cells [105]. Matrix cells are known to be a source of SHH, and secreted SHH regulates epithelial cell proliferation and dermal papilla maturation. Moreover, SHH governs dermal adipocyte precursors in adult hair cycling. In turn, dermal adipocytes and adipocyte precursors influence hair cycle progression [107,108,109]. In addition, dermal adipocytes secrete antimicrobial peptides to protect the skin from bacterial infection [110]. The roles of the HH signaling pathway in skin homeostasis are summarized in Figure 4.

## 8. Signaling Crosstalk between HH and Other Signaling Pathways in Epidermal Homeostasis

### 8.1. Wnt Signaling

The Wnt signaling pathway is also important for the maintenance of hair follicles in adult skin. As described above, activation of canonical Wnt signaling induces SHH expression, indicating HH signaling activation during hair follicle development in mouse embryos. Previous studies have demonstrated that the Wnt–HH signaling axis is also essential for maintaining the hair cycle. Epidermal basal cell-specific β-catenin activation in adult mouse skin also induces SHH expression, resulting in HH signaling activation [38,39], and HH signaling is required for β-catenin-mediated hair follicle formation [39].

### 8.2. The Hippo–YAP Signaling Pathway

The Hippo signaling pathway is a master regulator of cell proliferation and organ size. Hippo pathway molecules, such as macrophage-stimulating 1/2 (MST1/2) and large tumor suppressor 1/2 (LATS1/2), regulate a transcriptional coactivator, Yes-associated protein (YAP) [111,112]. YAP controls stem or progenitor cell proliferation in the mouse postnatal epidermis [113]. Furthermore, YAP functions in balancing growth and differentiation in the skin [114,115].

Akladios et al. proposed a novel interplay among HH, YAP, Rho-associated protein kinase (ROCK) and β-catenin in the control of skin homeostasis [116]. HH signaling induces YAP activation, and activated YAP in turn induces β-catenin activation. HH, YAP, and β-catenin together induce GLI2 activation to regulate epidermal homeostasis. At the same time, HH signaling activates ROCK in the epidermis. HH signaling and activated ROCK regulate cell proliferation and actin remodeling in the dermis.

### 8.3. p53/p63

The tumor suppressor, p53, is expressed in the epidermal basal layer and also in the outer root sheath and matrix of the hair follicles. Its expression is increased during the transition from anagen to the regression phase of the hair cycle (catagen), suggesting its role in hair follicle involution [117]. In addition, we previously demonstrated that HH signaling enhances the ubiquitin-mediated degradation of p53 through GLI1 [118]. Therefore, it is possible that HH signaling regulates hair cycling through regulating p53 protein.

A homolog of p53, p63, has an essential role in development. Mice lacking p63 show extensive defects in epidermal development and die at birth [119,120]. In epidermis, an anti-apoptotic protein and GLI target gene product, B-cell lymphoma 2 (BCL2) [121,122], is expressed in basal keratinocytes and the outer root sheath cells of the hair follicle. Its expression is associated with undifferentiated cells having proliferative potential. On the other hand, suprabasal keratinocytes do not express BCL2 [123]. These results suggest that BCL2 has an important role in regulating keratinocyte proliferation for skin homeostasis. Chari et al. demonstrated crosstalk between ΔNp63α, an isoform of p63 that is a crucial regulator of epidermal development, and HH signaling in keratinocytes [124]. HH signaling activation inhibits keratinocyte differentiation via GLI1-mediated upregulation of BCL2. At the same time, HH signaling activation results in ΔNp63α expression. Upregulated p63 then activates transcription in keratinocytes of a negative regulator of HH signaling, SUFU. In vivo analysis indicates that SUFU is also a negative regulator of the Wnt signaling pathway [125]. Overall, these results suggest that the HH–ΔNp63α axis regulates keratinocyte fate determination (proliferation or differentiation) for skin homeostasis.

## 9. Aberrant HH Signaling Pathway Activation and BCC

BCCs are so named because of their histological resemblance to basal cells of the interfollicular epidermis and epidermal appendages, that is, the keratinocytes adjacent to the stroma [126,127]. Activation of the HH signaling pathway through various mechanisms has been observed in BCCs [5]. Below, we introduce the mechanisms of BCC formation via the HH signaling pathway.

Although in general, BCCs seem to have relatively stable genomes, 67% of BCCs exhibit loss of *PTCH1*, 5% exhibit loss of *SUFU* and 10% have activating mutations of *SMO* [33,128,129]. However, GLI mutations (*GLI1*, *GLI2*, and *GLI3*) are not observed in BCC patients, although GLI overexpression is observed. In addition, *NRAS*, *KRAS*, *HRAS*, *BRAF*, and *CTNNB1* are not known to carry mutations in BCC patients [128].

Based on the molecular genetics analysis of human BCCs, several studies have been performed using genetically-modified mice in which the HH signaling pathway is constitutively activated. Constitutive or conditional overexpression of either GLI1 [130] or GLI2 [131] in keratinocytes can produce BCC-like lesions in the skin. Similarly, expression of mutant SMO carrying the activating mutations identified in human BCCs [33] can also produce murine BCCs. Furthermore, *Ptch1*^+/−^ mice develop BCCs, and those BCCs often exhibit a deletion of the wild-type copy of *Ptch1*, as well as up-regulation of HH signaling. Similarly, mice carrying one inactivated, mutant allele of the HH suppressor, *SUFU* are also susceptible to BCC development [132].

Although these results show that the HH signaling pathway has important roles in BCC formation, the actual cell of origin remains to be elucidated. X-ray-induced BCCs in *Ptch1*^+/−^ mice arise predominantly, and likely exclusively, from hair follicle bulge stem cells expressing keratin 15 (a bulge cell marker). Furthermore, upregulation of SMO expression is necessary for BCC formation in X-irradiated *Ptch1*^+/−^ mice [133]. Using an inducible mouse model enabling expression of a constitutively active SMO mutant (SMO-M2) [33] in the adult epidermis, HH pathway activation was found to reprogram adult interfollicular cells to an embryonic hair follicle progenitor fate. Furthermore, HH pathway activation induces the activation of Wnt/β-catenin signaling. Deletion of β-catenin in adult SMO-M2-expressing cells prevents reprogramming to an embryonic hair follicle progenitor fate and also prevents tumor initiation [134]. Recently, deletion of *Ptch1* in different cellular compartments revealed that tumors can arise from multiple hair follicle stem cells and from innervated GLI1-expressing progenitor cells in touch domes. In contrast, tumors do not arise from interfollicular epidermal stem cells [135].

BCC-specific signaling crosstalk between the HH and other signaling pathways has been reported. Eberl et al. reported crosstalk between the HH and EGFR signaling pathways in BCCs [41]. Skin-specific SMO-M2-expressing transgenic mice develop BCC-like lesions. However, skin-specific knockout of EGFR in SMO-M2-expressing mice reduces the number and size of BCCs. Furthermore, cooperation of HH and EGFR signaling induces specific GLI target genes such as *FGF19*, *TGFA*, *CXCR4*, *SOX9*, and *SOX2*. These genes are not induced by activation of the canonical HH signaling pathway alone. These results suggest that cooperation between HH and EGFR signaling is important for BCC development, which is mediated by HH–EGFR cooperation-responsive GLI target genes.

Vismodegib [6] is the first SMO inhibitor to receive approval from the USA FDA as a therapeutic for treating advanced or metastatic BCC. However, some studies have reported the emergence of SMO inhibitor-resistant tumors [136,137]. It will be important to understand the mechanisms by which SMO inhibitor-resistant cancer cells emerge, and to find novel therapeutic targets for these tumors. As a therapeutic strategy for SMO inhibitor-resistant tumors, several GLI1 inhibitors, such as JQ1 [138], Glabrescione B (GlaB) [139], and arsenic trioxide (ATO) [140], have been developed and tested using in vitro or in vivo models of BCC. Tang et al. demonstrated that bromodomain containing 4 (BRD4) directly occupies the *GLI1* and *GLI2* promoters and regulates *GLI1* and *GLI2* transcription downstream of SMO [139]. Treatment with JQ1, a small-molecule inhibitor targeting BRD4 [141], attenuates mouse BCC cell-derived tumor growth through the suppression of *GLI1* and *GLI2* transcription. In addition, JQ1-mediated downregulation of *GLI1* and *GLI2* transcription is also observed in SMO inhibitor-resistant BCC cells [138]. Infante et al. developed a novel GLI1 inhibitor (GlaB) that inhibits the interaction of GLI1 with DNA. They then demonstrated that GlaB inhibits GLI1-dependent BCC cell growth in vitro and BCC tumor growth in ASZ001 BCC allografts in vivo [139]. Ally et al. tried a combined treatment with ATO plus itraconazole for metastatic BCC patients [140]. Although tumor shrinkage was not observed, tumor growth was arrested for three months, and GLI1 mRNA levels were dramatically decreased. Improvement of the drug dose or dosing schedule may yield a better clinical response. Finally, we review another therapeutic target for SMO inhibitor-resistant BCC. Atwood et al. reported that atypical protein kinase Ci/λ (aPKC-i/λ) is a novel GLI1 target gene product, and a novel therapeutic target for SMO inhibitor-resistant BCC [142]. aPKC-i/λ functions downstream of SMO to phosphorylate and activate GLI1, resulting in maximal DNA binding and transcriptional activation. Activated aPKC-i/λ is upregulated in SMO inhibitor-resistant tumors, and targeting aPKC-i/λ suppresses GLI1 activation and the growth of SMO inhibitor-resistant BCC cell lines. These results show the potential of aPKC-i/λ as a therapeutic target for SMO inhibitor-resistant BCC.

## 10. Conclusions

The HH signaling pathway is one of the most fundamental in epidermal development and epidermal stem cell maintenance. However, deregulated HH signaling induces BCC. Hence, it is important that the intensity of the HH signal is strictly regulated during skin generation and homeostasis. To achieve precise regulation of HH signaling intensity, negative regulators of HH signaling are necessary, as well as crosstalk between the HH and other signal transduction pathways such as those activated by Wnt and BMP. Recently, it has been reported that low HH signaling activity is important for lung homeostasis [143]. Therefore, it is possible that fine-tuning the intensity of a HH signal may be involved in the homeostasis of multiple organs, including the skin.

As described above, aberrant activation of HH signaling causes BCC formation. A tumor microenvironment would exhibit not only the HH pathway regulatory mechanisms that are common with skin development and homeostasis, but also cancer cell-specific regulatory mechanisms. Therefore, understanding the precise mechanisms of HH pathway regulation in the tumor microenvironment is important to advance new therapeutic strategies for SMO inhibitor-resistant BCC. Moreover, such knowledge may also help to provide new therapeutic strategies for other HH pathway-activated cancers.
